# Survival kinase genes present prognostic significance in glioblastoma

**DOI:** 10.18632/oncotarget.7917

**Published:** 2016-03-04

**Authors:** Robin T. Varghese, Yanping Liang, Ting Guan, Christopher T. Franck, Deborah F. Kelly, Zhi Sheng

**Affiliations:** ^1^ Virginia Tech Carilion Research Institute, Roanoke, VA 24016, USA; ^2^ Laboratory for Interdisciplinary Statistical Analysis, Department of Statistics, Virginia Tech, Blacksburg, VA 24061, USA; ^3^ Department of Biological Sciences, College of Sciences at Virginia Tech, Blacksburg, VA 24061, USA; ^4^ Department of Internal Medicine, Virginia Tech Carilion School of Medicine, Roanoke, VA 24016, USA; ^5^ Department of Biological Sciences and Pathobiology, Virginia-Maryland College of Veterinary Medicine, Virginia Tech, Blacksburg, VA 24061, USA; ^6^ Faculty of Health Science, Virginia Tech, Blacksburg, VA 24061, USA

**Keywords:** survival kinase genes, glioblastoma, tumor recurrence, prognosis, PIK3CB

## Abstract

Cancer biomarkers with a strong predictive power for diagnosis/prognosis and a potential to be therapeutic targets have not yet been fully established. Here we employed a loss-of-function screen in glioblastoma (GBM), an infiltrative brain tumor with a dismal prognosis, and identified 20 survival kinase genes (SKGs). Survival analyses using The Cancer Genome Atlas (TCGA) datasets revealed that the expression of CDCP1, CDKL5, CSNK1E, IRAK3, LATS2, PRKAA1, STK3, TBRG4, and ULK4 stratified GBM prognosis with or without temozolomide (TMZ) treatment as a covariate. For the first time, we found that GBM patients with a high level of NEK9 and PIK3CB had a greater chance of having recurrent tumors. The expression of CDCP1, IGF2R, IRAK3, LATS2, PIK3CB, ULK4, or VRK1 in primary GBM tumors was associated with recurrence-related prognosis. Notably, the level of PIK3CB in recurrent tumors was much higher than that in newly diagnosed ones. Congruent with these results, genes in the PI3K/AKT pathway showed a significantly strong correlation with recurrence rate, further highlighting the pivotal role of PIK3CB in the disease progression. Importantly, 17 SKGs together presented a novel GBM prognostic signature. SKGs identified herein are associated with recurrence rate and present prognostic significance in GBM, thereby becoming attractive therapeutic targets.

## INTRODUCTION

Glioblastoma (GBM) is the most common and deadly subtype of malignant brain tumors [1]. Although the incidence of GBM is low (0.59–3.69 per 100,000 persons worldwide), its clinical outcome has been extremely poor despite aggressive upfront treatments including maximum safe surgical removal of the tumor, ionized irradiation, and chemotherapeutic treatment using temozolomide (TMZ) [2]. The five-year overall survival (OS) of GBM is approximately 4.7% in the United States [2] and even below this rate in Europe [3]. The median survival of GBM patients receiving aforementioned concurrent therapies is only 14.6 months [4]. These grim facts therefore demonstrate an urgent need of new and effective treatments as well as powerful prognostic markers to assist these treatments for this deadly disease.

Prognosis markers are important for GBM treatments such as chemotherapy temozolomide (TMZ). For example, promoter methylation status of the O6-methylguanine-DNA methyltransferase (MGMT)––an enzyme that repairs TMZ-induced DNA damage––predicts the response of GBM patients to this drug [2, 5, 6]. In several clinical trials, MGMT methylation was confirmed as an important prognosis marker associated with improved OS of newly diagnosed GBM patients receiving TMZ [6–12]. However, given the limited therapeutic efficacy of TMZ, the importance of MGMT as a standard prognostic marker for GBM is compromised. There are microRNAs and some other genes such as epidermal growth factor receptor (EGFR) or CD133 that show a close correlation with GBM prognosis [13–17]. However, targeting them has not yet been very successful. Recent advances in large-scale genome DNA sequencing have significantly contributed to the identification of new biomarkers for GBM [18, 19]. For instance, genetic mutations in isocitrate dehydrogenase 1 or 2 (IDH1/2) genes have shown a strong association with better outcome in a subgroup of GBM [20, 21]. An inhibitor of IDH1 significantly retards GBM growth through inducing differentiation [22]. However, this genetic approach often lacks functional information of candidate genes thereby requiring a further extensive and time-consuming investigation.

Loss-of-function screening using a library of short hairpin RNAs (shRNAs) [23–29] provides a useful platform that allows a search of candidate genes for cancer therapeutic development based on their functions/activities. Yet this approach has not been used to identify genes that can serve as both therapeutic targets and prognostic markers. In this report, we employed a loss-of-function screen using a library of shRNAs against human kinases and identified 20 kinases critical for the survival of human U87MG GBM cells. Further analyses revealed candidate kinases, whose expression correlated with the prognosis of newly diagnosed and/or recurrent GBM. In addition, we for the first time have revealed kinase genes with a strong association with recurrence rate.

## RESULTS

### A loss-of-function screen identifies new kinases essential for the survival of human U87MG GBM cells

**Figure 1:**
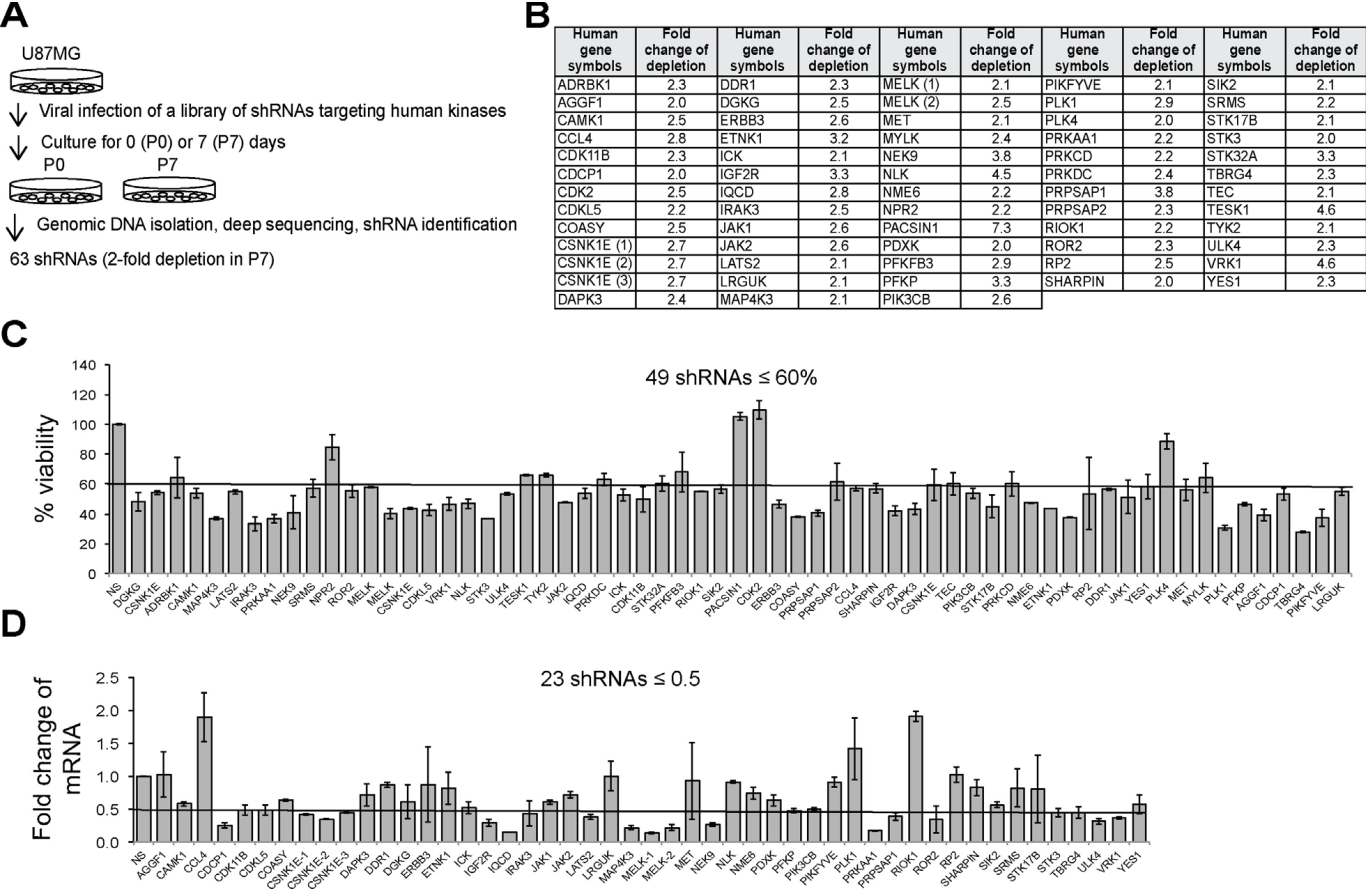
A loss-of-function screen identifies SKGs in U87MG cells (**A**) A diagram illustrating the loss-of-function screen. In principle, a short hairpin (sh) RNA of a potential SKG that is depleted overtime is under-represented in P7, compared to P0. (**B**) Candidate SKGs with at least 2-fold reduction of shRNA sequence copies in P7 compared to those in P0. (**C**) Viability assay. 63 individual shRNAs of SKGs were introduced into U87MG cells. The cell viability was measured using the MTS cell viability assay. Candidate SKG shRNAs were normalized to non-silencing (NS) shRNA. The cutoff line is 60%. (**D**) Knockdown efficiency. The knockdown of SKGs by their shRNAs was assessed using the quantitative RT-PCR. The cut-off line is 2-fold reduction.

To identify genes with a potential to be new biomarkers and therapeutic targets, we focused on kinase genes that often govern cancer cell survival. To identify these kinases, we employed a loss-of-function screen (outlined in Figure 1A) that utilizes a library of shRNAs targeting 781 human kinase genes. We divided the human U87MG GBM cells transduced with viruses harboring the above library of shRNAs into two parts: one part was collected immediately as passage 0 (P0); the other part continued a 7-day culture and was saved as passage 7 (P7). The genomic DNA was then isolated and DNA libraries containing PCR-amplified shRNA sequences were prepared. By using Solexa deep sequencing, we profiled shRNAs. In principle, an shRNA that causes a drastic growth inhibition would be depleted from the cell population and has a decreased sequencing read number at P7. We found that 63 shRNAs had 2-fold less of the sequencing read number at P7 compared to P0 (Figure 1B). We further validated these candidates as follows. 49 individual candidate shRNAs decreased the viability of U87MG cells to ≤ 60%, compared to those with the control non-silencing (NS) shRNA (Figure 1C). 23 out of 49 shRNAs caused a ≥ 2-fold reduction of mRNAs of their target genes (Figure 1D). As there were multiple shRNAs against CSNK1E or MELK, 23 shRNAs targeted 20 kinase genes that are important for the survival of U87MG cells. Full names of 20 candidate kinase genes were listed in Table 1. We termed these 20 candidates as survival kinase genes (SKGs). Among these SKGs, MELK has been previously reported as a survival gene in GBM [30].

**Table 1:** Survival kinase genes

Gene Symbol	Gene full name
CDCP1	CUB Domain Containing Protein 1
CDK11B	Cyclin-Dependent Kinase 11B
CDKL5	Cyclin-Dependent Kinase-Like 5
CSNK1E	Casein Kinase 1, Epsilon
IGF2R	Insulin-Like Growth Factor 2 Receptor
IQCD	IQ Motif Containing D
IRAK3	Interleukin-1 Receptor-Associated Kinase 3
LATS2	Large Tumor Suppressor Kinase 2
MAP4K3	Mitogen-Activated Protein Kinase Kinase Kinase Kinase 3
MELK	Maternal Embryonic Leucine Zipper Kinase
NEK9	NIMA-Related Kinase 9
PFKP	Phosphofructokinase, Platelet
PIK3CB	Phosphatidylinositol-4, 5-Bisphosphate 3-Kinase, Catalytic Subunit Beta
PRKAA1	Protein Kinase, AMP-Activated, Alpha 1 Catalytic Subunit
PRPSAP1	Phosphoribosyl Pyrophosphate Synthetase-Associated Protein 1
ROR2	Receptor Tyrosine Kinase-Like Orphan Receptor 2
STK3	Serine/Threonine Kinase 3
TBRG4	Transforming Growth Factor Beta Regulator 4
ULK4	Unc-51 Like Kinase 4
VRK1	Vaccinia Related Kinase 1

### SKGs are enriched in GBM

**Figure 2:**
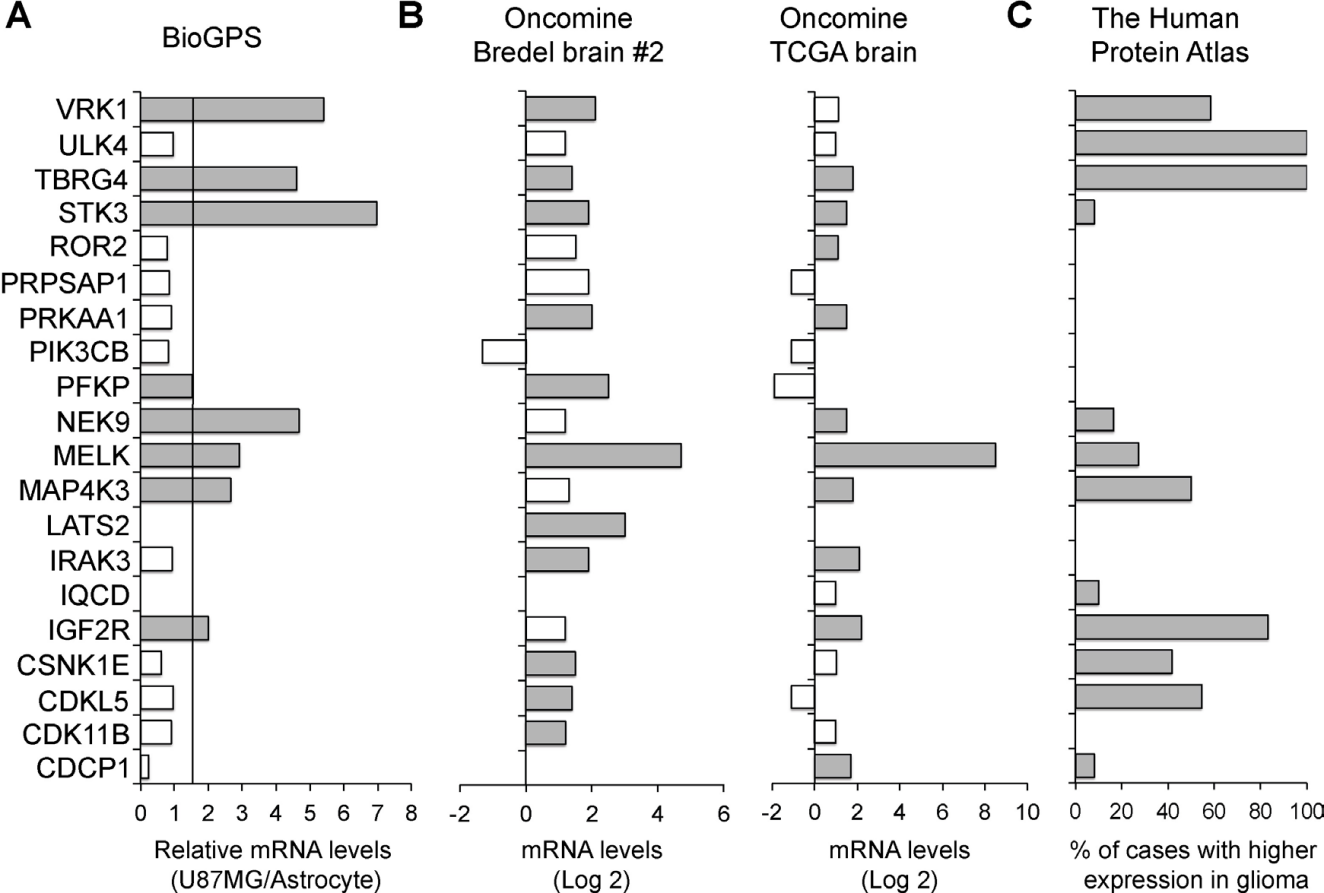
SKGs are enriched in GBM (**A**) mRNA levels of SKGs in U87MG. Gene expression data was retrieved from the BioGPS database. Fold changes of SKG mRNAs in U87MG cells compared to those in astrocytes were shown. The cut-off line was 1.5-fold increase. SKGs with a high level of mRNA in U87MG cells were labeled as grey bars. (**B**) mRNA levels of SKGs in glioblastoma tissues. Gene expression data was retrieved from the Oncomine database. The mRNA levels of SKGs in glioblastoma were compared to those in normal brain tissues. Results from two studies (Bredel brain #2 and TCGA brain) were shown. *P < 0.05. SKGs with a statistically significant increase of mRNA in glioblastoma in either study were labeled as grey bars. (**C**) Protein levels of SKGs in glioma. Protein expression data was retrieved from the Human Protein Atlas database. The percentages of glioma cases with a higher level of SKGs compared to the normal brain were shown. SKGs with more protein in glioma were labeled as grey bars. PRPSAP1 and PIK3CB showed a negative result in all these analyses.

We then tested the hypothesis that SKGs are enriched in GBM due to their essentiality in GBM survival. We queried online gene expression databases such as BioGPS [31], Oncomine (ThermoFisher Scientific), and The Human Protein Atlas [32]. We found that mRNA levels of 8 SKGs (IGF2R, MAP4K3, MELK, NEK9, PFKP, STK3, TBRG4, and VRK1) were at least 1.5-fold higher in U87MG cells than those in astrocytes based on BioGPS (Figure 2A, grey bars). We also analyzed two GBM datasets (Bredel Brain #2 and TCGA brain) in Oncomine. 17 SKGs (except IQCD, PIK3CB, and PRPSAP1) showed statistically higher levels (*P* < 0.05) of their mRNAs in GBM tissues when compared to normal brain tissues (Figure 2B, grey bars). Furthermore, we compared the protein levels of SKGs between glioma and normal brain. There were more than 8% of cases with a higher protein level of 12 SKGs (CDCP1, CDKL5, CSNK1E, IGF2R, IQCD, MAP4K3, MELK, NEK9, STK3, TBRG4, ULK4, and VRK1) in glioma (Figure 2C, grey bars). Together, these results demonstrate that SKGs are enriched in GBM.

### Expression of SKGs correlates with the OS of GBM patients

**Figure 3:**
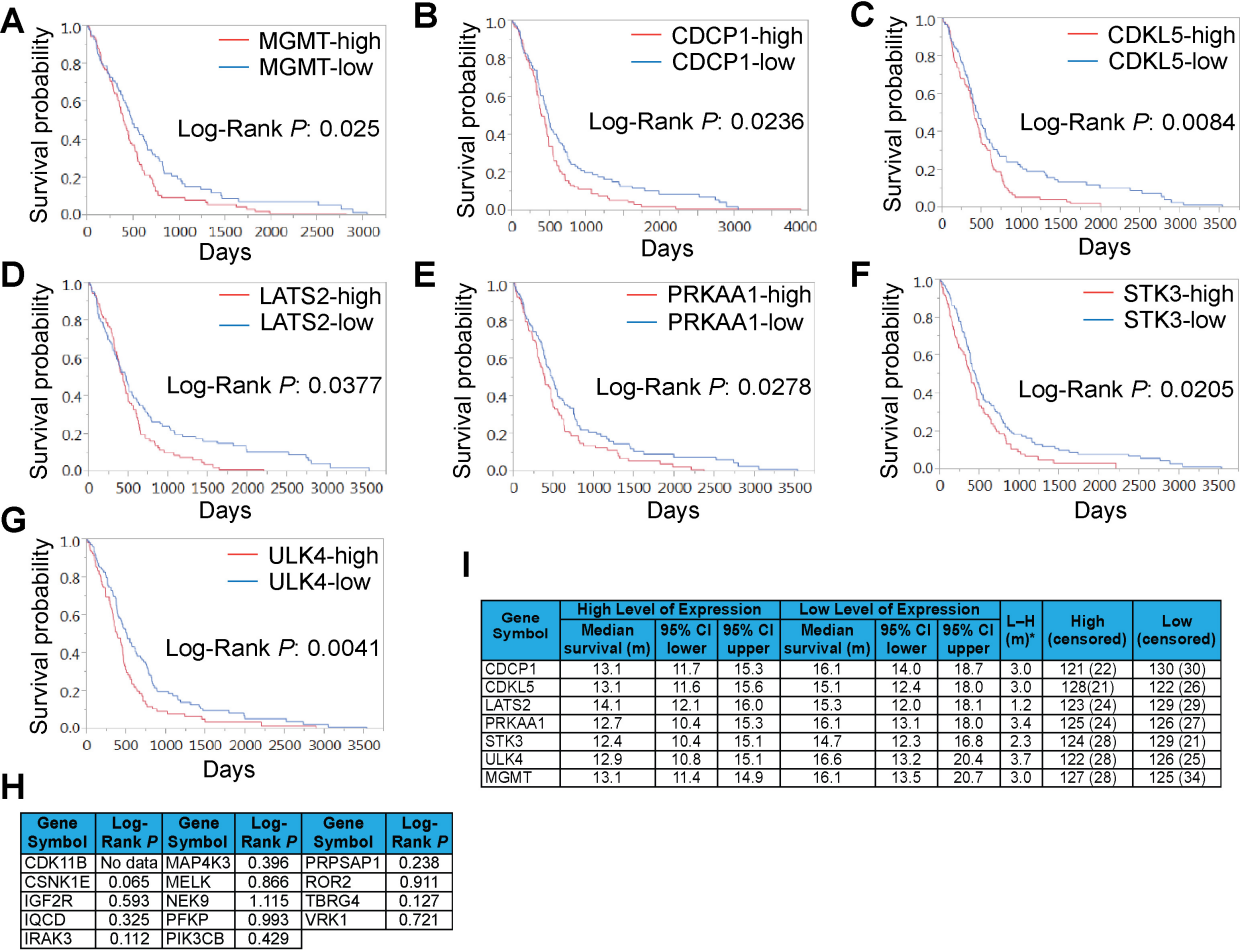
Expression of SKGs correlates with GBM prognosis The Kaplan Meier analysis was performed using the TCGA GBM datasets. The survival curves of MGMT (**A**), CDCP1 (**B**), CDKL5 (**C**), LATS2 (**D**), PRKAA1 (**E**), STK3 (**F**), and ULK4 (**G**) with Log-Rank *P* values were shown. The Log-Rank *P* values of other SKGs that showed no statistical significance were listed in (**H**). The media survival time of prognostic SKGs was shown in (**I**).

The above results prompted us to evaluate the potential of SKGs as GBM prognostic markers. We retrieved the gene expression data of initial/primary GBM tumors and the corresponding patient information associated with these tumors from TCGA [33] (http://cancergenome.nih.gov/). The MGMT mRNA level (used as a positive control) showed an inverse correlation with the OS of GBM (Figure 3A), congruent with previous reports [6, 8]. We then analyzed 20 SKGs using the Kaplan Meier (KM) survival analysis. We found that the expression of CDCP1, CDKL5, LATS2, PRKAA1, STK3, and ULK4 was adversely associated with the OS of GBM and their Log-Rank *P* values were less than 0.05 or 0.01 (Figure 3B–3G). Other SKGs showed no statistical significance (Figure 3H and Supplementary Figure S1). There was no expression data pertaining to CDK11B in this cohort of GBM patients (Figure 3H). Patients with low levels of the above 6 SKGs lived 1.2 to 3.7 months longer than those with high levels of SKGs (Figure 3I). In contrast, patients with less MGMT lived three month longer. Hence, CDCP1, CDKL5, LATS2, PRKAA1, STK3, and ULK4 present prognostic significance in the OS of GBM.

We next utilized a Cox proportional hazard model to test whether TMZ treatment or age could be used as a covariate with SKGs to better measure GBM prognosis. TMZ and age have been associated with GBM clinical outcomes [4, 34]. The hazard ratios (HRs, which define the death risk) of SKGs with prognostic significance were equivalent to or higher than that of MGMT, whereas SKGs with no prognostic significance had a lower HRs (Table 2, Cox Univariate and highlighted in grey), verifying the results of KM survival analysis (Figure 3B–3H). When TMZ––but not age––was used as a covariate, HRs of prognostic SKGs (except CDKL5) increased (Table 2, comparing Cox Univarate with Cox Multivariate with TMZ). Intriguingly, IRAK3 and TBRG4 showed a significant inverse correlation (*P* < 0.05) with the OS of GBM only when TMZ was used as a covariate (Table 2, highlighted in grey), whereas the expression of CSNK1E was positively associated with GBM prognosis (Table 2, highlighted in grey). Thus, the Cox multivariate analysis with TMZ reveals three more SKGs with prognostic significance. However, the analysis of Cox multivariate with age revealed that only CDCP1 and CDKL5 showed statistical significance (Table 2, Cox multivariate with age). These results suggest that SKGs and TMZ (but not age) together show a better prognostic correlation.

**Table 2:** Survival analysis of SKGs using the cox proportional hazard model

	Cox Univariate				Cox Multivariate with TMZ				Cox Multivariate with Age			
Gene Symbol	HR (H vs L)	95% CI lower	95% CI upper	Log-Rank *p*	HR (H vs L)	95% CI lower	95% CI upper	Log-Rank *p*	HR (H vs L)	95% CI lower	95% CI upper	Log-Rank *p*
CDCP1	1.382	1.042	1.832	0.025	1.384	1.040	1.842	0.026	1.332	1.006	1.764	0.045
CDK11B	No Data											
CDKL5	1.467	1.102	1.957	0.009	1.441	1.074	1.935	0.015	1.368	1.027	1.827	0.032
CSNK1E	0.762	0.569	1.017	0.065	0.684	0.507	0.921	0.012	0.954	0.708	1.282	0.755
IGF2R	0.927	0.702	1.224	0.594	0.804	0.598	1.081	0.150	1.025	0.774	1.356	0.865
IQCD	1.152	0.868	1.527	0.326	1.226	0.918	1.637	0.167	1.077	0.810	1.432	0.607
IRAK3	1.255	0.947	1.661	0.113	1.416	1.056	1.898	0.020	1.050	0.790	1.396	0.735
LATS2	1.356	1.016	1.810	0.039	1.411	1.048	1.903	0.023	1.057	0.790	1.417	0.710
MAP4K3	1.127	0.854	1.483	0.397	1.274	0.960	1.686	0.093	1.042	0.789	1.373	0.772
MELK	1.024	0.775	1.354	0.866	1.125	0.843	1.503	0.425	0.989	0.748	1.309	0.941
NEK9	1.166	0.875	1.552	0.293	1.344	0.991	1.821	0.057	1.056	0.792	1.407	0.708
PFKP	0.999	0.749	1.333	0.993	0.854	0.633	1.150	0.298	1.191	0.887	1.600	0.245
PIK3CB	1.119	0.845	1.478	0.431	1.160	0.869	1.543	0.313	0.918	0.690	1.219	0.555
PRKAA1	1.372	1.034	1.822	0.029	1.499	1.120	2.006	0.006	1.227	0.923	1.633	0.159
PRPSAP1	1.185	0.893	1.573	0.239	1.229	0.916	1.648	0.169	1.174	0.884	1.558	0.266
ROR2	1.016	0.766	1.353	0.911	1.078	0.805	1.449	0.616	1.138	0.854	1.522	0.379
STK3	1.393	1.050	1.848	0.022	1.558	1.166	2.078	0.003	1.096	0.817	1.471	0.540
TBRG4	1.245	0.939	1.650	0.128	1.399	1.042	1.880	0.026	1.179	0.889	1.565	0.254
ULK4	1.518	1.139	2.023	0.005	1.550	1.153	2.080	0.004	1.244	0.926	1.671	0.147
VRK1	0.950	0.717	1.260	0.722	1.050	0.785	1.407	0.743	0.976	0.737	1.295	0.864
MGMT	1.390	1.040	1.858	0.026	1.406	1.045	1.893	0.024	1.369	1.023	1.833	0.034

Data was retrieved from TCGA. HR (H vs L): Hazard ratios comparing the group with high (H) levels of SKGs to the one with low (L) levels. CI: confidence interval. Log-Rank *P* values less than 0.05 were highlighted in grey.

### Expression of SKGs correlates with the incidence rate and prognosis of recurrent GBMs

**Figure 4:**
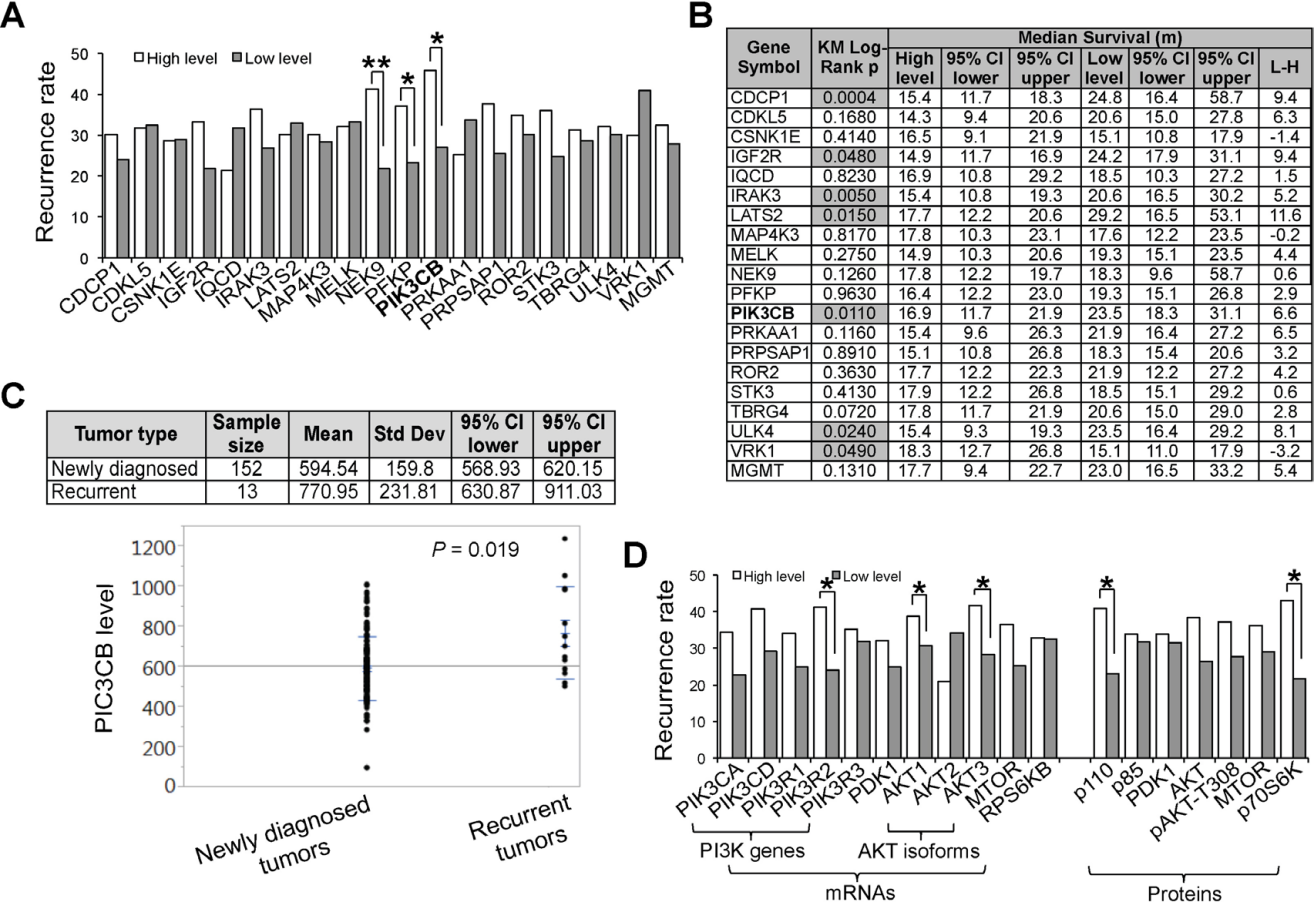
Expression of SKGs correlates with the incidence rate and prognosis of recurrent GBMs (**A**) Recurrence rate. GBM recurrence rates in patients with a high level (white bars) or a low level of SKGs (grey bars) were shown. The statistical difference between two groups was determined by the Fisher's exact test. (**B**) KM survival analysis. The relationship of SKGs with the prognosis of GBMs patients with recurrent tumors was analyzed. The KM Log-Rank *P* values were shown. The Log-Rank *P* values less than 0.05 were highlighted in grey. VRK1 showed an effect on patient survival opposite to that exhibited by other SKGs. Median survival time of high- or low-level groups together with the lower or upper 95% CI (confidence interval) was listed. (**C**) Expression of PIK3CB in newly diagnosed and recurrent GBM tumors. RNA-seq data were retrieved from the TCGA database. The mean values of PIK3CB transcripts were shown. (**D**) GBM recurrence and PI3K/AKT pathway. Kinases involved in or downstream of PI3K/AKT pathway were analyzed. *P < 0.05; **P < 0.01.

Nearly 90% of GBM patients experience tumor recurrence two years after treatments [35]. Often patients with a recurrent tumor can not undergo a second major neurosurgery and resist radiation and chemotherapy [35]. Thus, identifying recurrence-related SKGs would help in the early diagnosis of and perhaps treatment for recurrence. To determine the role of SKGs in tumor recurrence, we first selected GBM patients with disease progression information including post-treatment recurrence data. Note that the sample size was small (from 17 to 33) due to the limited information regarding recurrence (Supplementary Table S1). We first analyzed the recurrence rate in GBM patients with a high or low level of SKGs. The high levels of 14 SKGs were associated with a greater incidence of recurrence (Figure 4A and Supplementary Table S1). However, only NEK9 (*P* < 0.01), PFKP (*P* < 0.05), and PIK3CB (*P* < 0.05) showed a statistically significant difference, highlighting their potential in predicting GBM recurrence rate. Further KM survival analysis revealed that the Log-Rank *P* values of CDCP1, IGF2R, IRAK3, LATS2, PIK3CB, ULK4, and VRK1 were < 0.05 (Figure 4B and Supplementary Figure S2), suggesting that the expression of these SGKs in the newly diagnosed GBM has a statistically significant correlation with the patient survival associated with recurrence. Notably, CDCP1, IRAK3, LATS2 and ULK4 also presented prognostic significance in the OS of GBM (Figure 3 and Table 2). The difference in median survival time ranged from 5.2 to 11.6 months (Figure 4B), in stark contrast to the maximum 3.7 months of GBMs' OS (Figure 3I). These results demonstrate a pivotal role of SKGs in GBM recurrence. As an exception, the low level of VRK1 was associated with a shorter life span of GBM recurrence (Figure 4B). By contrast, MGMT and PTEN failed to show a significant correlation with either recurrence rate (Figure 4A and Supplementary Table S1) or recurrence-associated survival (Figure 4B and Supplementary Figure S2). Taken together, we have revealed new biomarkers for the prognosis of GBM patients with recurrent tumors.

### PI3K/AKT pathway is associated with the incidence rate and prognosis of GBM recurrence

Among SKGs associated with GBM recurrence, one intriguing gene is PIK3CB, a candidate SKG that showed a strong correlation with both recurrence rate and prognosis (Figure 4A and 4B). Consistent with these results, the mRNA levels of PIK3CB were significantly higher (*P =* 0.019) in recurrent tumors than those in newly diagnosed GBM tumors (Figure 4C). PIK3CB is a catalytic subunit of PI3K complex [36]. The PI3K/AKT pathway has been implicated in GBM recurrence and its inhibitors have been used to treat recurrent tumors [37, 38]. We then analyzed multiple kinases in or downstream of this pathway. GBM patients expressing high levels of kinases in the PI3K/AKT pathway had a greater chance of recurrence (Figure 4D, panel mRNAs). Among these genes, PIK3R1, AKT1, and AKT3 showed a statistical significance (*P* < 0.05). We also analyzed the protein levels of these kinases using the TCGA Reverse Phase Protein Array dataset. The high protein level was associated with a greater chance of tumor recurrence (Figure 4D, panel Proteins). Notably, the PI3K regulatory subunit PI3K p85 and an MTOR target p70S6K showed a statistical significance (*P* < 0.05). Collectively, our results demonstrate that the PI3K/AKT signaling is involved in the disease progression, highlighting the importance of PIK3CB as a predictor of recurrence rate and/or a prognosis marker associated with recurrence.

### A group of SKGs presents a novel prognostic signature for GBM

**Figure 5:**
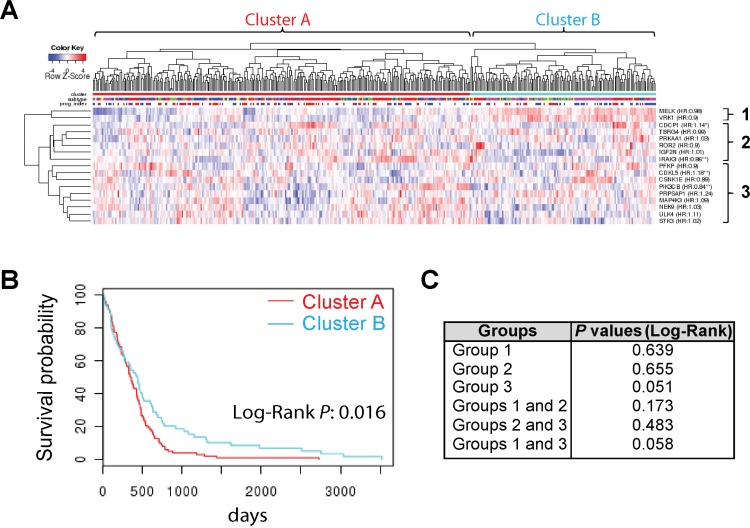
A group of SKGs presents a novel prognostic signature for GBM (**A**) GBM patient clustering using the GBM Bio Discovery Portal. (**B**) KM survival analysis. (**C**) Log-Rank *P* values of KM survival analysis of different subgroups or combinations were listed.

To further verify the role of SKGs in GBM prognosis, we analyzed the prognostic potential of multiple SKGs as a group. We employed an online program Glioblastoma Bio Discovery Portal (GBM-BioDP) that exploits different computer algorithms to cluster genes together based on their expression profile in the TCGA GBM datasets [39]. Three SKGs (CDK11B, IQCD, and PRKAA1) were not included in this analysis due to the lack of information in all datasets. Based on the expression profile of the remaining 17 SKGs, GBM patients were divided into cluster A and B (Figure 5A). Cluster A showed a significantly shorter life span than cluster B with a Log-Rank *P* at 0.016 (Figure 5B). The clustering analysis also identified three subgroups (Figure 5A). We then analyzed the relationship of SKGs in these subgroups with GBM survival by dividing GBM patients into clusters (Supplementary Figure S3) followed by KM survival analysis (Figure 5C and Supplementary Figure S4). None of these subgroups presented a significant correlation with GBM survival. Together, our data strongly suggests that a group of 17 SKGs presents a novel signature for GBM prognosis.

## DISCUSSION

The results presented herein are of importance due to their significance in measuring the prognosis of GBM and/or tumor recurrence as well as the druggablilty of these SKGs. Based on the literature (Supplementary Table S2), 10 SKGs has been reported previously as a prognosis biomarker for certain types of cancer. However, none of SKGs has been used as a prognosis marker for GBM. Our finding that SKGs can predict GBM prognosis is therefore novel and unprecedented. Interestingly, the low rather than high levels of CSNK1E, IGF2R, IRAK3, and PRKAA1 relate to poor cancer prognosis (Supplementary Table S2), suggesting that kinases often play a divergent role in different types of cancer. By contrast, the high expression levels of most prognostic SKGs (except CSNK1E) show a correlation with the poor prognosis of GBM patients (Table 2). Hence, inhibiting these prognostic SKGs represents a viable approach to developing new therapies for GBM.

Our study also reveals powerful biomarkers for the diagnosis and/or prognosis of tumor recurrence, given the fact that no similar markers have been established to date except magnetic resonance imaging (MRI) [40]. MRI is a costly approach often perplexed with pseudo-progression [41]. Measuring the gene expression levels in tumor biopsies would provide an effective and less expensive way to monitor disease progression. Particularly important is that the high levels of NEK9, PFKP, and PIK3CB in the newly diagnosed GBM tumors are associated with the greater risk of recurrence. Thus, NEK, PFKP, and PIK3CB can be used as a predictive marker for GBM recurrence rate, highlighting a potentially important approach for an early diagnosis.

The identification of PIK3CB as a marker for predicting GBM recurrence rate and prognosis sets up stage for future development of diagnostic tools and therapies for this disease. In fact, targeting PI3K has been an effort in the clinic to treat GBM. However, the therapeutic benefits are limited. For instance, a recent phase II trial of PX-866 (a pan PI3K inhibitor) failed to yield promising therapeutic effects and demonstrated no association of PI3KCA with disease progression [38]. Based on our findings, it would be imperative to further investigate the possibility of targeting PIK3CB specifically, rather than the PI3K pathway that has more profound impact on cell fate. Interestingly, specific and clinically applicable inhibitors of PIK3CB (i.e. AZD8186 [42] and GSK2636771) are currently available [43], presenting a potential opportunity for clinical trials to treat newly diagnosed or recurrent GBM.

## METHODS

### Reagents

The Cell-Titer 96^®^ Aqueous One solution cell proliferation assay (MTS) was purchased from Promega. The SYBER green mix was purchased from Promega. The TRC kinase shRNA gene family library was purchased from GE Dharmacon. The Column-free^™^ plasmid mini-prep kit was purchased from Lamda Biotech, Inc. The QIAamp DNA extraction kit was purchased from QIAGEN. Puromycin was purchased from Thermo Fisher Scientific. Trizol and the SuperScript^®^ III RT were purchased from Thermo Fisher Scientific.

### Cell culture

U87MG cells were maintained in Dulbecco's modified Eagle medium (Thermo Fisher Scientific) supplemented with 10% fetal bovine serum (FBS) (Atlas Biologicals, Inc.), streptomycin (100 μg/ml) and penicillin (100 IU/ml) (Thermo Fisher Scientific). Cells were cultured in a 37°C incubator with 5% CO_2_.

### shRNA library preparation

4,518 shRNA constructs (against 781 human kinases) were maintained as a single clone of bacteria in glycerol stock in 96-well plates. To prepare a mix of plasmids, each bacterial plate was replicated in another 96-well culture plate with 1.8 ml of 2 × LB in each well. Plates were then incubated at 37°C with vigorous shaking for 24 hours. Plasmids were then prepared using a mini-prep kit adapted for 96-well plate. The concentration of each plasmid was determined using a nanodrop (Thermo Fisher Scientific). Equal amount of ∼450 plasmids was mixed together as a single plasmid pool. The entire library was then divided into 10 pools.

### Lentivirus preparation

The TRC shRNAs are built upon pLKO.1 vector that can be used to generate lentivirus. A pool of plasmids of the TRC kinase shRNA library was transfected into HEK293T cells together with packaging plasmids pMD2.g and psPax2. 48 hours later, the culture media that contained lentiviruses was collected and divided into small aliquots. Aliquots were stored at −80°C freezer. The virus titer was then determined using the serial dilution assay.

### Loss-of-function screen for SKGs

U87MG (1 × 10^6^) cells were transduced with 10 pools of lentiviruses that harbor the TRC kinase shRNAs. One day after viral infection, half of the infected cells (5 × 10^5^) was collected as P0 (initial time point) and subjected to genomic DNA isolation described below. The other half of infected cells was cultured for another 7 days in the media containing 0.5 μg/ml puromycin and collected as P7 (end time point). Genomic DNA of cells at P0 and P7 was isolated using the QIAamp DNA extraction kit. shRNAs were then amplified by PCR (MF18 (5′-tacgatacaaggctgttagagag-3′) and MF19 (5′-cgaaccgcaaggaaccttc-3′)) and sequenced using the Solexa deep sequencing. The sequencing read number of each shRNA at P0 was divided by that at P7. The shRNAs with a 2-fold less of this number were considered as candidate SKGs.

### Cell viability assay

To validate the primary candidates identified from the above screen, U87MG cells were transduced with viruses of NS or individual candidate shRNAs. Cells were then selected with puromycin (0.5 μg/ml) for a week. Cell viability was determined using the MTS assay described previously [44, 45].

### Quantitative RT-PCR

Quantitative RT-PCR was preformed as described previously [44, 45]. U87MG cells transduced with NS or candidate shRNAs were subject total RNA extraction using Trizol. RNA was quantified using a nanodrop. 2 μg of total RNA was used to prepare cDNA using the SuperScript^®^III RT. mRNA levels of shRNA-targeting kinase genes were quantified using a real-time PCR assay.

### Analysis of gene expression

The expression data of 20 SKGs was retrieved from BioGPS, Oncomine, and The Human Protein Atlas. For the data from BioGPS, the relative level of each SKG mRNA in GBM cell lines was obtained by dividing the arbitrary number of SKGs in tumor cell lines with that in astrocytes. The fold changes of each SKG in normal brain tissues or glioblastoma were retrieved from Oncomine. The *p* values determining the difference of SKG levels between normal brain and glioblastoma were also retrieved from the database. To analyze the protein expression data from the Human Protein Atlas, the level of SKG proteins (low, medium, high) in normal cerebral cortex was used as the reference. Glioma samples with the SKG protein level higher than the reference were scored. The enrichment of SKG protein in glioma was presented as percentages of glioma cases with a higher level of SKG protein.

### GBM patient survival analyses

GBM patient survival analyses were performed as previously described [31]. In brief, clinical variables of glioblastoma patients, such as survival time, vital status, disease progression, TMZ treatment, and tumor recurrence, were retrieved from the TCGA (The Cancer Genome Atlas) Data Portal (https://tcga-data.nci.nih.gov/tcga/). Gene expression data, including for glioblastoma patients (AgilentG4502A071; AgilentG4502A072), was downloaded from the Pan-cancer project (syn1461183) on Synapse (http://www.synapse.org). GBM patients were divided into high level (top 25%) and low level (bottom 25%) based on the SKG mRNA levels. A Kaplan Meier analysis or a Cox proportional hazard model was performed using the JMP software (SAS Institute Inc.). Recurrent tumor rates were predicted using Contingency Analysis and Fisher's Exact Test was performed using the JMP software.

### GBM discovery bio portal

To analyze the expression of SKGs as groups, we employed the online program GBM Discovery Bio Portal that utilizes the same TCGA GBM datasets described above. The algorithms used to cluster GBM patients include: [1] The optimal number of clusters (NbClust); [2] Expression levels in GBM subgroups; [3] Prognostic Index, obtained by computing weighted averages of expression values with regression coefficients of a multi-gene Cox proportional hazards model. The datasets used herein include: [1] AgilentG4502A_07 from University of North Carolina, [2] HT_HG-U133A from Broad Institute, and [3] HuEx-1_0-st-v2 from the Berkeley Lab.

## SUPPLEMENTARY MATERIALS FIGURES AND TABLES


