# Supplementary material for: Survival kinase genes present prognostic significance in glioblastoma
Source: Oncotarget. 2016 Mar 4;7(15):20140–51. doi: 10.18632/oncotarget.7917 (PMC4991443; doi:10.18632/oncotarget.7917)
Supplement: Supplementary file 1 [file oncotarget-07-20140-s001.pdf]

Survival kinase genes present prognostic significance in glioblastoma

Supplementary Materials

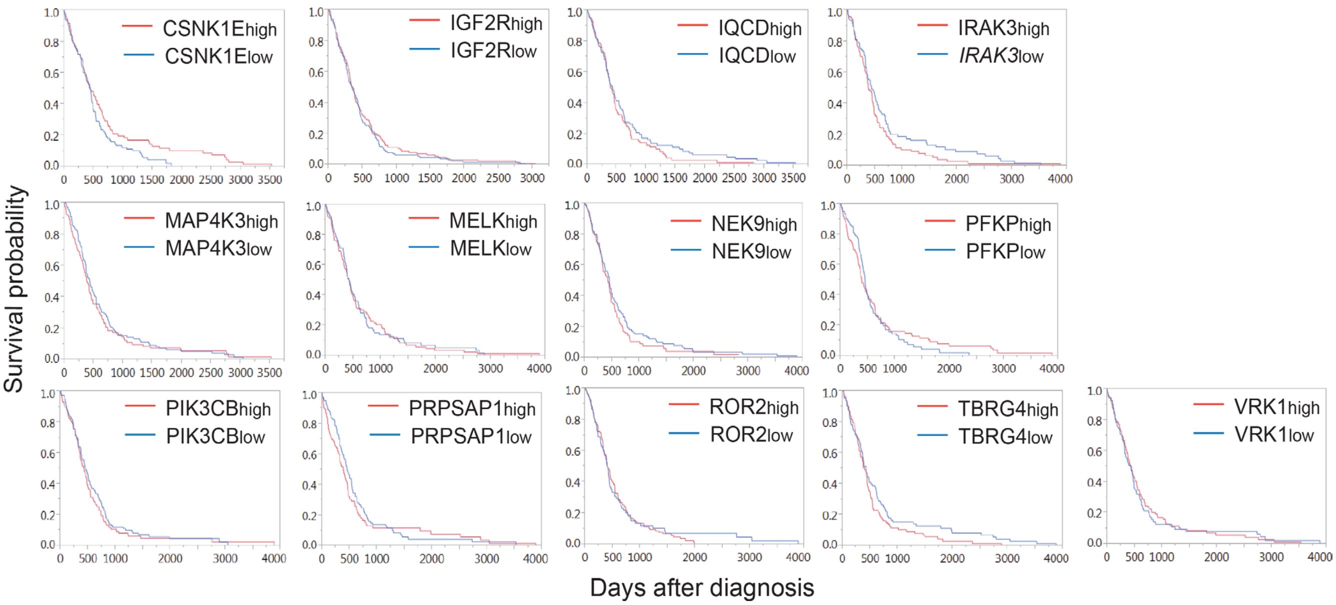

Supplementary Figure S1: Kaplan Meier survival analysis of SKGs with no statistical significance. The survival analysis results of SKGs with no statistical significance were shown.

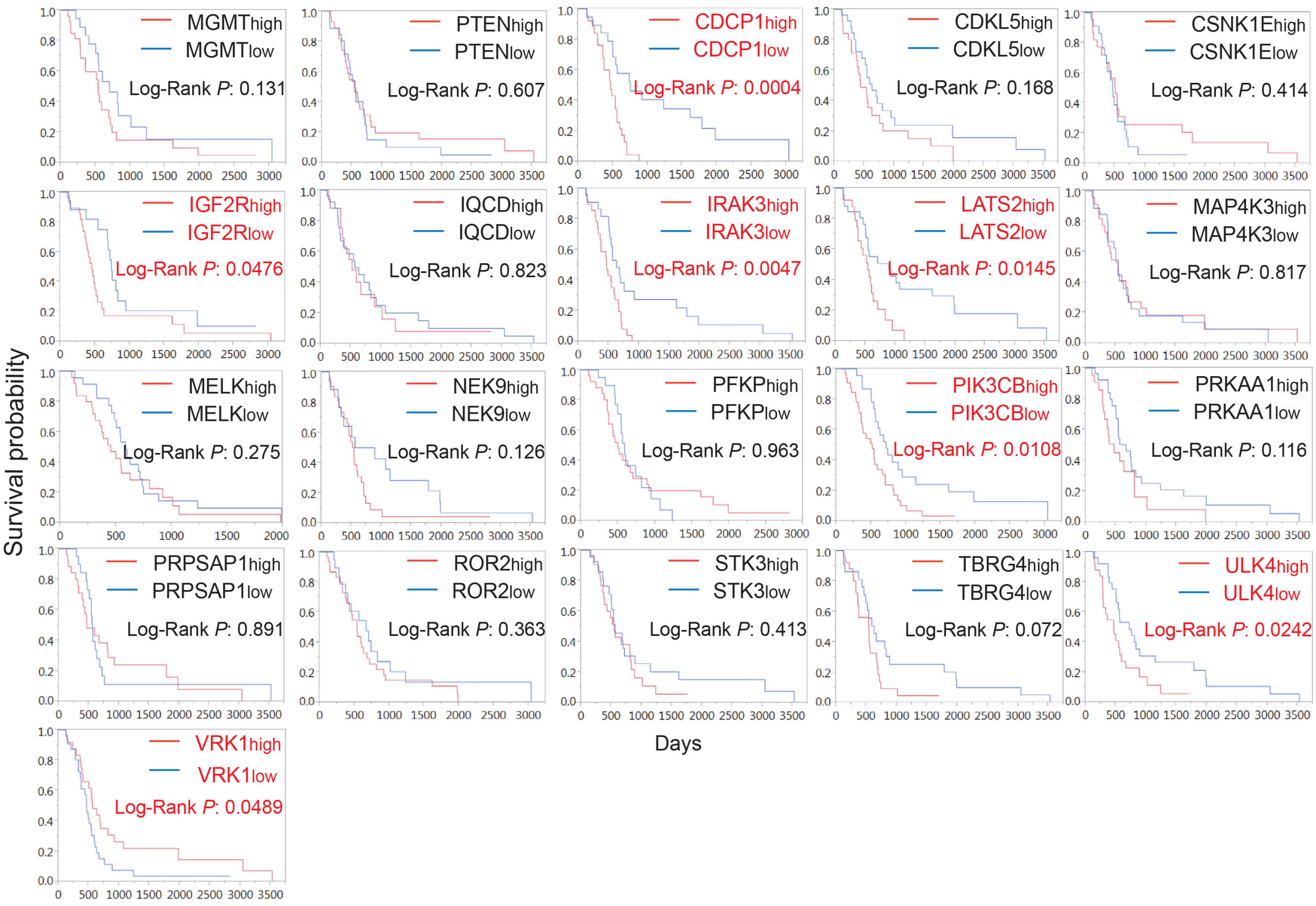

Supplementary Figure S2: SKGs and the survival of recurrent GBMs. SKGs with a statistical significance in the survival of recurrent GBMs were highlighted in red. MGMT and PTEN were also shown.

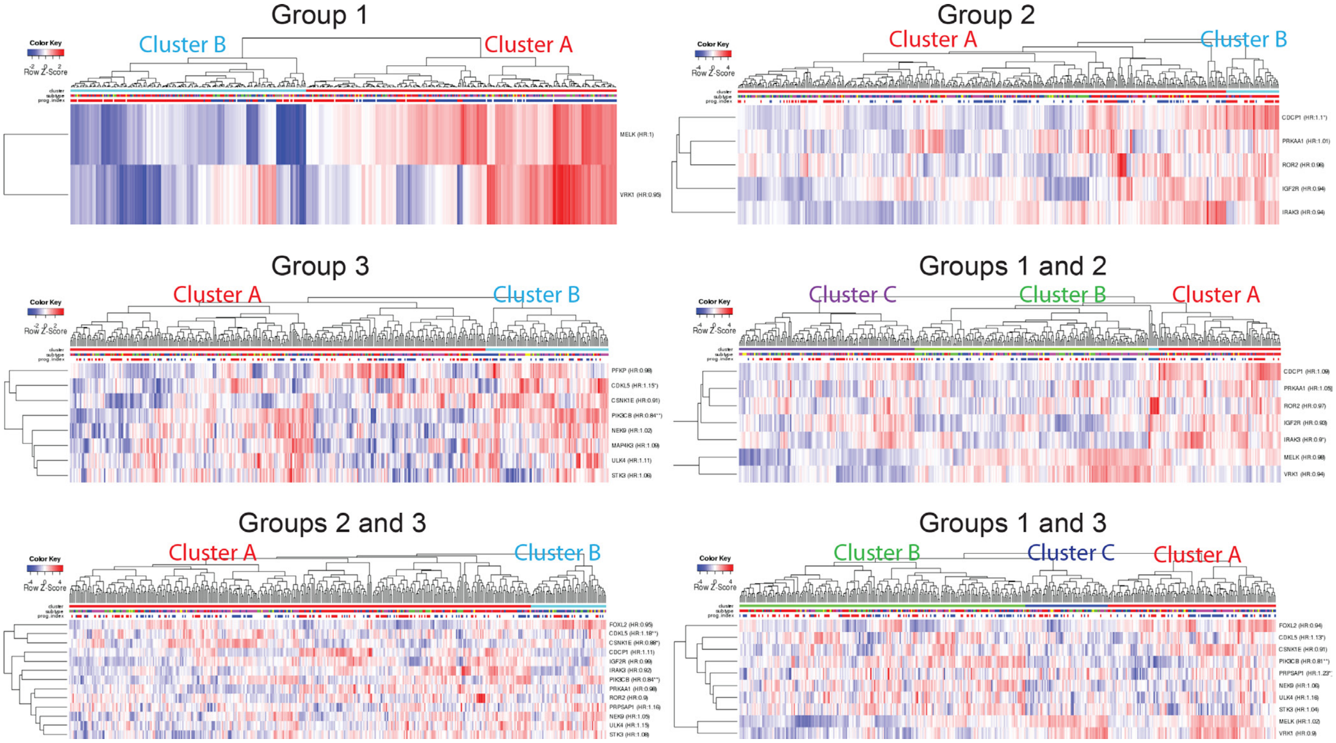

Supplementary Figure S3: Clustering of GBM patients based on the gene expression profile of SKGs. GBM datasets from the TCGA database were used. 6 different groups of SKGs were analyzed.

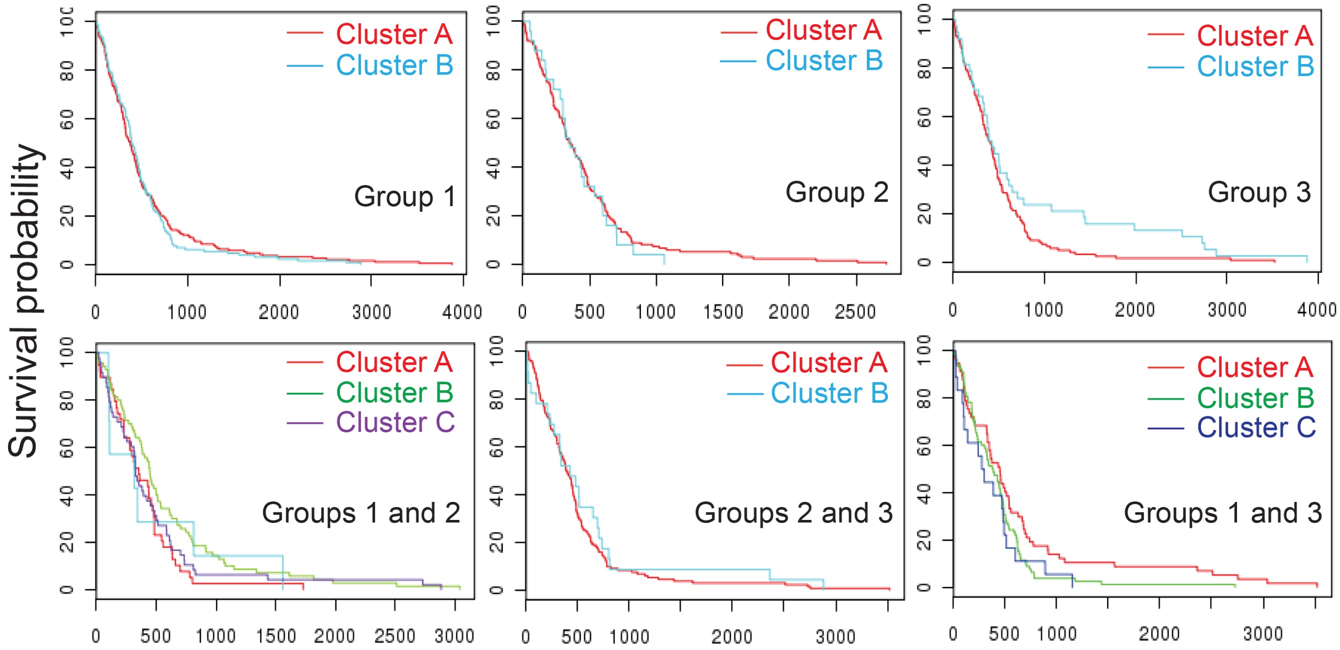

Supplementary Figure S4: Groups of SKGs and GBM survival. GBM datasets from the TCGA database were used for survival analysis. GBM patients were clustered based on the expression profile of SKGs and subject to Kaplan Meier analysis. No statistically significant difference was found.

Supplementary Table S1: SKGs and GBM recurrence

Gene Symbol	Recurrence Rate						
	High level	Nrecur	Ntotal	Low level	Nrecur	Ntotal	*p* (High vs Low)
CDCP1	30.2%	26	86	24.1%	19	79	0.237
CDKL5	31.7%	25	79	32.5%	26	80	0.612
CSNK1E	28.7%	22	78	29.0%	22	76	0.61
IGF2R	33.3%	29	87	21.8%	17	78	0.07
IQCD	21.3%	17	80	31.7%	26	82	0.954
IRAK3	36.4%	28	77	26.8%	22	82	0.131
LATS2	30.1%	25	83	32.9%	26	79	0.709
MAP4K3	30.1%	27	73	28.3%	26	92	0.463
MELK	32.1%	25	78	33.3%	24	72	0.634
NEK9	41.3%	31	75	21.8%	17	78	0.007
PFKP	37.1%	26	70	23.3%	20	86	0.043
PIK3CB	45.8%	33	72	27.1%	23	85	0.011
PRKAA1	25.3%	21	83	33.8%	26	77	0.911
PRPSAP1	37.7%	26	69	25.6%	22	86	0.075
ROR2	34.9%	30	86	30.2%	19	63	0.335
STK3	36.0%	27	75	24.7%	22	89	0.081
TBRG4	31.3%	26	83	28.6%	22	77	0.418
ULK4	32.1%	25	78	30.1%	25	83	0.462
VRK1	30.0%	24	80	41.0%	32	787	0.947
MGMT	32.5%	27	83	27.8%	20	72	0.321
PTEN	35.0%	28	80	31.7%	26	82	0.391

The GBM patients with disease progression information were first divided into two groups based on the expression levels of SKGs (high level vs low level). The GBM recurrence rates were percentages of cases with a recurrent tumor (Nrecur) over the total cases (Ntotal). The statistical difference between the high level group and the low level one was determined using the Fisher's exact test.

Supplementary Table S2: SKGs and cancer prognosis

Gene Symbol	Effect of SKGs on prognosis	Cancer type (Reference*)
CDCP1	High level → poor prognosis.	Ovarian cancer (1); Breast cancer (2); Colorectal cancer (3)
	Low level → poor prognosis.	Esophageal cancer (4)
CDK11B	No reports	
CDKL5	No reports	
CSNK1E	Low level → poor prognosis.	Colerectal cancer (5); Oral cancer (6)
IGF2R	Low level → poor prognosis.	Lung cancer (7); Hepatocellular carcinoma (7); Head and neck cancer (8)
IQCD	No reports	
IRAK3	Low level → poor prognosis.	Hepatocellular carcinooma (9)
LATS2	High level → poor prognosis.	Nasopharyngeal carcinoma (10)
MAP4K3	No reports	
MELK	High level → poor prognosis.	Lung cancer (11); Breast cancer (12, 13); Prostate cancer (14)
NEK9	No reports	
PFKP	No reports	
PIK3CB	High level → poor prognosis.	Rectal carcinoma (15); Colorectal cancer (16); Diffuse large B cell lymphoma (17); Breast cancer (18)
PRKAA1	Low level → poor prognosis.	Colorectal cancer (19); Melanoma (20); Non-Hodgkin lymphoma (21); Ovarian cancer (22)
PRPSAP 1	No reports	
ROR2	High level → poor prognosis.	Cervical cancer (23); Colorectal cancer (24); Breast cancer (25); Gastrointestinal stromal tumor (26); Osteosarcoma (27)
STK3	No reports	
TBRG4	No reports	
ULK4	No reports	
VRK1	High level → poor prognosis.	Breast cancer (28)

REFERENCES

1 He Y, *et al*. Elevated CDCP1 predicts poor patient outcome and mediates ovarian clear cell carcinoma by promoting tumor spheroid formation, cell migration and chemoresistance. Oncogene, doi:10. 1038/onc. 2015. 101.

2 Alajati A, *et al*. Interaction of CDCP1 with HER2 enhances HER2-driven tumorigenesis and promotes trastuzumab resistance in breast cancer. Cell reports 11, 564–576, doi:10. 1016/j. celrep. 2015. 03. 044.

3 Chou CT, *et al*. Prognostic Significance of CDCP1 Expression in Colorectal Cancer and Effect of Its Inhibition on Invasion and Migration. Annals of surgical oncology, doi:10. 1245/s10434–015–4505–4.

4 Tiong KL, *et al*. CSNK1E/CTNNB1 Are Synthetic Lethal To TP53 in Colorectal Cancer and Are Markers for Prognosis. Neoplasia 16, 441–450, doi:10. 1016/j. neo. 2014. 04. 007.

5 Lin SH, *et al*. Casein kinase 1 epsilon expression predicts poorer prognosis in low T-stage oral cancer patients. Int J Mol Sci 15, 2876–2891, doi:10. 3390/ijms15022876.

6 Tian Z, Yao G, Song H, Zhou Y, Geng J. IGF2R expression is associated with the chemotherapy response and prognosis of patients with advanced NSCLC. Cell Physiol Biochem 34, 1578–1588, doi:10. 1159/000366361.

7 Jang HS, *et al*. Clinical significance of loss of heterozygosity for M6P/IGF2R in patients with primary hepatocellular carcinoma. World journal of gastroenterology: WJG 14, 1394–1398.

8 Jamieson TA, *et al*. M6P/IGF2R loss of heterozygosity in head and neck cancer associated with poor patient prognosis. BMC Cancer 3, 4.

9 Kuo CC, *et al*. Methylation of IRAK3 is a novel prognostic marker in hepatocellular carcinoma. World journal of gastroenterology: WJG 21, 3960–3969, doi:10. 3748/wjg. v21. i13. 3960.

10 Zhang Y, *et al*. LATS2 is de-methylated and overexpressed in nasopharyngeal carcinoma and predicts poor prognosis. BMC Cancer 10, 538, doi:10. 1186/1471–2407–10–538.

11 Li Y, *et al*. Network-based approach identified cell cycle genes as predictor of overall survival in lung adenocarcinoma patients. Lung Cancer 80, 91–98, doi:10. 1016/j. lungcan. 2012. 12. 022.

12 Pickard MR, *et al*. Dysregulated expression of Fau and MELK is associated with poor prognosis in breast cancer. Breast Cancer Res 11, R60, doi:10. 1186/bcr2350.

13 Ross-Adams H, *et al*. Integration of copy number and transcriptomics provides risk stratification in prostate cancer: A discovery and validation cohort study. EBioMedicine 2, 1133–1144, doi:10. 1016/j. ebiom. 2015. 07. 017.

14 Liu R, Guo C.X, Zhou H.H. Network-based approach to identify prognostic biomarkers for estrogen receptor-positive breast cancer treatment with tamoxifen. Cancer Biol Ther 16, 317–324, doi:10. 1080/15384047. 2014. 1002360.

15 Wen F, He S, Sun C, Li T, Wu S. PIK3CA and PIK3CB expression and relationship with multidrug resistance in colorectal carcinoma. International journal of clinical and experimental pathology 7, 82958303.

16 Yu WD, *et al*. Phosphatidylinositol 3-kinase CB association with preoperative radiotherapy response in rectal adenocarcinoma. World journal of gastroenterology: WJG 20, 16258–16267, doi:10. 3748/wjg. v20. i43. 16258.

17 Cui W, *et al*. Frequent copy number variations of PI3K/AKT pathway and aberrant protein expressions of PI3K subunits are associated with inferior survival in diffuse large B cell lymphoma. J Transl Med 12, 10, doi:10. 1186/1479–5876–12–10.

18 Carvalho S, Milanezi F, Costa JL, Amendoeira I, Schmitt F. PIKing the right isoform: the emergent role of the p110beta subunit in breast cancer. Virchows Archiv: an international journal of pathology 456, 235243, doi:10. 1007/s00428–010–0881–0.

19 Lee SJ, *et al*. Genetic variations in STK11, PRKAA1, and TSC1 associated with prognosis for patients with colorectal cancer. Annals of surgical oncology 21 Suppl 4, S634–639, doi:10. 1245/s10434–014–3729-z.

20 Bhandaru M, Martinka M, Li G, Rotte A. Loss of AMPKalpha1 expression is associated with poor survival in melanoma patients. J Invest Dermatol 134, 1763–1766, doi:10. 1038/jid. 2014. 26.

21 Hoffman AE, Demanelis K, Fu A, Zheng T, Zhu Y. Association of AMP-activated protein kinase with risk and progression of non-Hodgkin lymphoma. Cancer Epidemiol Biomarkers Prev 22, 736–744, doi:10. 1158/1055–9965. EPI-12–1014.

22 Li C, Liu VW, Chiu PM, Chan D.W, Ngan H.Y. Over-expressions of AMPK subunits in ovarian carcinomas with significant clinical implications. BMC Cancer 12, 357, doi:10. 1186/1471–2407–12–357.

23 Sun B, Ye X, Lin L, Shen M, Jiang T. Up-regulation of ROR2 is associated with unfavorable prognosis and tumor progression in cervical cancer. International journal of clinical and experimental pathology 8, 856–861.

24 Mei H, *et al*. High expression of ROR2 in cancer cell correlates with unfavorable prognosis in colorectal cancer. Biochem Biophys Res Commun 453, 703–709, doi:10. 1016/j. bbrc. 2014. 09. 141.

25 Henry C, *et al*. Expression of the novel Wnt receptor ROR2 is increased in breast cancer and may regulate both beta-catenin dependent and independent Wnt signalling. Journal of cancer research and clinical oncology 141, 243–254, doi:10. 1007/s00432–014–1824-y.

26 Edris B, *et al*. ROR2 is a novel prognostic biomarker and a potential therapeutic target in leiomyosarcoma and gastrointestinal stromal tumour. The Journal of pathology 227, 223–233, doi:10. 1002/path. 3986.

27 Lu BJ, *et al*. Expression of WNT-5a and ROR2 correlates with disease severity in osteosarcoma. Molecular medicine reports 5, 1033–1036, doi:10. 3892/mmr. 2012. 772.

28 Martin KJ, Patrick DR, Bissell MJ, Fournier M.V. Prognostic breast cancer signature identified from 3D culture model accurately predicts clinical outcome across independent datasets. PLoS One 3, e2994, doi:10. 1371/journal. pone. 0002994.
